# Extracellular Vesicles-Dependent Secretion Regulates Intracellular CYFIP2 Protein Homeostasis in Cortical Neurons

**DOI:** 10.3390/biomedicines13102518

**Published:** 2025-10-15

**Authors:** Michael J. Culp, Breandan J. Rosolia, Cameron Keyser, Jingqi Yan

**Affiliations:** 1 Center for Gene Regulation in Heath and Disease, Department of Biological, Geological, and Environmental Sciences, Cleveland State University, Cleveland, OH 44115, USAb.rosolia@vikes.csuohio.edu (B.J.R.);; 2 Department of Neuroscience, Lerner Research Institute, Cleveland Clinic, Cleveland, OH 44195, USA

**Keywords:** extracellular vesicles, CYFIP2, synapse, neurons, Fragile X Syndrome

## Abstract

**Background**: Fragile X Syndrome (FXS) is the most common monogenic cause of autism spectrum disorders, and is characterized by the excessive immature excitatory synapses in cortical neurons, leading to excitatory/inhibitory imbalance and core autistic behaviors. This synaptic pathology has been attributed to dysregulated levels of synaptic proteins, including CYFIP2: a key regulator of synaptic structure and plasticity. However, the mechanism underlying the increased CYFIP2 protein level in FXS neurons remains unclear. Neurons abundantly secrete extracellular vesicles (EVs) enriched with bioactive cargos (proteins and miRNAs). **Objectives**: the goal of this research is to identify whether EV-dependent secretion plays important roles in regulating the intracellular CYFIP2 protein level in WT and FXS neurons. **Methods and Results**: our proteomic analysis reveals that CYFIP2 protein is packaged in EVs released by mouse cortical neurons. Pharmacological and genetic blockades of neuronal EV release significantly elevated intracellular CYFIP2 levels by 78 ± 14% and 168 ± 39%, respectively. Glutamate-evoked EV release significantly reduced the CYFIP2 level by 24 ± 2%. Neurons from *Fmr1* KO mice, an FXS model, secreted significantly less EVs (46 ± 5%) than the wild type, and showed significantly elevated CYFIP2 (by 155 ± 31%). Evoking EV release in FXS neurons significantly lowered the intracellular CYFIP2 (by 53 ± 6%). **Conclusions**: these findings identify an EV-secretion-dependent mechanism that controls neuronal CYFIP2 level, implicating EV-mediated export in the regulation of synaptic protein homeostasis, synaptic remodeling, and FXS-associated synaptic deficits.

## 1. Introduction

Fragile X Syndrome (FXS) is an inherited genetic disorder linked to intellectual disabilities and is the most common monogenic cause of autism spectrum disorders (ASDs) [1,2,3]. FXS arises from an enlargement of a trinucleotide (CGG) repeat >200 times in the 5′ Untranslated Region (5′ UTR) of the *Fragile X-Messenger Ribonucleoprotein 1* (*Fmr1*) gene [1,3]. The expansion induces the hypermethylated 5′ UTR and promoter region of *Fmr1,* resulting in transcriptional silencing and loss of the protein product, Fragile X-Messenger Ribonucleoprotein (FMRP), an RNA-binding protein [1,3]. Dendritic spines on neurons are the postsynaptic compartments receiving most of the excitatory signals [4]. Overabundance of dendritic spines with immature morphology is a hallmark neuropathological feature in FXS brain [1,3], which leads to imbalanced excitatory/inhibitory signals and core autistic behaviors, such as hypersensitivity and social deficits. Thus, investigations on the molecular mechanisms underlying the aberrant synapses may shed new light on new therapeutic strategies for synaptic and behavioral deficits in FXS [5,6].

Synaptic proteins play critical roles in regulating synapse stability, morphology, and functions [7,8,9]. Cytoskeleton filamentous actin (F-actin) is the primary structural support of synapses [10]. Brains from FXS patients show increased levels of Cytoplasmic *FMR1*-Interacting Protein 2 (CYFIP2), a highly conserved and crucial cytoplasmic synaptic protein [11]. CYFIP2 interacts with FMRP [12,13,14,15] and serves as a key component of the WAVE regulatory complex [16,17], which controls actin polymerization and thus influences synaptic structure, plasticity, and neuronal function [18,19]. CYFIP2 and its paralog, CYFIP1, are both enriched at excitatory synapses [20]. Hippocampal neurons with overexpressed CYFIP2 show increased density of dendritic spines with long and immature morphology [20], similar to FXS neurons, suggesting that the elevated CYFIP2 may be causally related to overabundance of immature excitatory synapses in FXS. Unlike the well-studied CYFIP1 [21,22,23,24], the functions and regulation of CYFIP2 are not yet fully understood. Importantly, although CYFIP2 protein levels are increased in cells of FXS patients, its mRNA level is not significantly changed [11]. Thus, revealing the mechanism underlying increased CYFIP2 protein in FXS neurons may explore a new pathway in regulating synaptic protein homeostasis and identify new therapeutic targets for FXS.

Extracellular vesicles (EVs) are nanosized particles derived from the endosomal membrane and released by all kinds of cells [25,26,27,28]. Small EVs (diameter: 30–150 nm), such as exosomes, are crucial mediators of intracellular communication within the brain, trafficking proteins, lipids, miRNAs, and RNAs [25,26,27,28,29]. EVs are generated through inward budding of the multivesicular body membrane (MVB) to form intraluminal vesicles (ILVs), which are released into the extracellular space after the fusion of MVB with the plasma membrane [25,26,27,28]. Synaptic proteins (PSD-95, Arc and Synaptotagmin), key regulators of synaptic organization and plasticity, are released through neuronal EVs and transported between neurons [30,31,32]. In addition, other EV protein cargos, such as HDAC2 and Wnt, also show strong regulatory effects on cytoskeleton organization and synaptic morphology of recipient neurons [33,34,35]. Thus, proteins capable of regulating synaptic structure and number are sorted into EVs and secreted by neurons.

Our initial proteomic analysis revealed that CYFIP2 protein is contained in neuronal EVs. Thus, the aim of this study is to further determine whether EV-mediated secretion of CYFIP2 protein is a key mechanism regulating intracellular CYFIP2 level in neurons, and to explore how EV secretion may contribute to the elevated CYFIP2 level observed in FXS neurons. To this end, we first examined the effects of the inhibition and activation of EV release on the intracellular CYFIP2 protein level in neurons. Furthermore, we compared EV release capabilities of neurons from the wild-type and FXS mice. Our findings indicated that suppression of EV release with a chemical inhibitor or knock out of *RAB27a*, a critical protein for EV release, both significantly increased CYFIP2 protein level in neurons. Glutamate-induced stimulation of EV release significantly reduced CYFIP2 level in neurons. Neurons from *Fmr1* KO mice, an FXS mouse model, secreted significantly less EVs than wild-type neurons, and showed increased intracellular CYFIP2 protein. Elevating EV release from *Fmr1* KO neurons effectively reduced the intracellular CYFIP2 level. These results identify a previously unrecognized EV release-dependent mechanism that controls the intracellular CYFIP2 level, implicating a role of EV secretion in regulating synaptic remodeling and FXS-related synaptic deficits.

## 2. Materials and Methods

*Animals.* Mice (FVB.129P2-Pde6b^+^ Tyr^c-ch^/AntJ (WT) and FVB.129P2-Pde6b^+^ Tyr^c-ch^ *Fmr1*^tm1Cgr^/J (*Fmr1* KO)) from the Jackson Laboratory were bred and maintained as described before [36]. *Rab27a* KO mice were created by collaborating with the Institute of Genomic Medicine of Texas A&M University through inserting a *lacZ sequence*, a *neo* gene, and two *Frt* sequences between exon2 and exon3 of the *Rab27a* gene of WT mice. The animal facility is pathogen-free, and operates on a 12 h/12 h light and dark cycle. Tail tissue biopsy and PCR were used for genotyping. Primers used for genotyping of *Rab27a* KO mice are: *Rab27a*-Forward, 5″-ACGGTTACCTACTGAATCATCTCC-3′; *Rab27a*-KO-Reverse, 5′-AACATAAAGTGACCCTCCCAACA-3′; and *Rab27a*-WT-Reverse, 5′-AGCCAAGAATGTATAAGTCCCTG-3′.

*Cell culture*. Primary cortical neurons dissected from embryonic 18 (E18) mice were cultured in a medium consisting of Neurobasal A (Gibco, Waltham, MA, USA), supplemented with 2% B-27 (Gibco) and 1% GlutaMAX (Gibco), as described before [36]. At day in vitro 7 (DIV7), the culture medium was changed, and neurons were treated as described in the “Study Design”.

*Immunocytochemistry*. Neurons cultured on poly-L-lysine (Sigma-Aldrich, St. Louis MO, USA) coated coverslips were washed 3 times with PBS, fixed with 4% paraformaldehyde (PFA), and blocked with 5% normal goat serum (Vector Laboratories, Newark, CA, USA) for one hour. Neurons were then incubated with primary antibodies overnight at 4 °C, followed by secondary antibodies conjugated with Alexa Fluor 488, 555, or 647 (Thermo Scientific, Waltham, MA, USA). After incubation with the secondary antibodies, neurons were washed and mounted with the VECTASHIELD Antifade Mounting Media with DAPI (Vector Laboratories). Use of DAPI staining revealed all cells. The Nikon confocal microscope (60× objectives) (Melville, NY, USA) was used for image acquisition. For measurement, multiple coverslips and areas per coverslip were selected randomly. Neurons used for quantification were chosen randomly. Image J software (NIH, 1.54g, Besthesda, MD, USA) was used for analysis. Microscope laser settings were uniform across all preparations. To ensure comparability, the same staining procedure was used.

*EV collection, analysis and imaging*. Neuronal EVs were collected as described before [37]. Culture medium was collected into 50 mL tubes and centrifuged at 2000× *g* for 10 min at 4 °C to remove dead cells. After being filtered through a 0.22 µm sterile filter, the medium was centrifuged at 4000× *g* for 30 min at 4 °C. The collected supernatant was then ultracentrifuged at 23,500 RPM for 110 min at 4 °C, using the Beckman Coulter Optima L-90 k Ultracentrifuge (Beckman Coulter, Brea, CA, USA) with the Beckman Coulter SW32Ti (Beckman Coulter). The pellet EVs were resuspended with PBS and ultracentrifuged in the same setting for the second time as washing. The supernatant was carefully discarded, and the final pellet EVs were resuspended with 100 µL PBS. Collected EVs were then subjected to nanoparticle tracking analysis (Zetaview, Particle Matrix, Inning am Ammersee, Germany) in the Flowcytometry Core, Cleveland Clinic. EVs were imaged with negative staining and electron microscopy, as described before [37]. Grids with EVs were imaged and examined in a FEI Tecnai G2 Spirit BioTWIN TEM (Imaging Core, Cleveland Clinics) (Thermo Scientific). A magnification of 30,000 was used.

*Protein lysate preparation and Western blot*. On DIV8, the neuron medium was removed and 100 µL RIPA lysis buffer (Thermo Scientific), supplemented with 1 µL Halt proteinase inhibitor cocktail (Thermo Scientific), was added to neurons. Lysates were sonicated (Fisherbrand, Waltham, MA, USA) and then centrifuged at 12,000 RPM for 5 min at 4 °C. The supernatant was collected, and protein concentrations were measured with the BCA kit (Thermo Scientific). For collection of EV proteins, EVs as the ultracentrifuge pellet were directly resuspended with RIPA lysis buffer (Thermo Scientific) supplemented with proteinase inhibitors (Sigma, St. Louis, MO, USA), and then chilled on ice for 30 min. Western blots were performed as described [36]. PVDF membranes (Millipore, Burlington, MA, USA) were imaged using the Li-Cor Odyssey system (Lincoln, NE, USA). Image J (NIH) software was used for the quantification of band intensities.

*Antibodies*. Primary antibodies used for immunofluorescence include rabbit anti-CYFIP2 (GeneTex, Irvine, CA, USA), chicken anti-MAP2 (Millipore), mouse anti-CD81 (Santa Cruz, Dallas, TX, USA), and mouse anti-CD63 (Santa Cruz). Secondary antibodies used for immunofluorescence include Alexa Fluor 488, 555, and 647 conjugated secondary antibodies (Thermo Scientific). Primary antibodies for the Western blot include rabbit anti-β-actin (Sigma), rabbit anti-CYFIP2 (GeneTex), mouse anti-CD81 (Santa Cruz), mouse anti-CD63 (Santa Cruz), and rabbit anti-Rab27a (Cell Signaling, Danvers, MA, USA). Secondary antibodies used for the Western blot are purchased from Invitrogen.

*Cell Viability Assay.* The MTT assay was performed according to the manual [38]. Neurons were incubated in 1 mg/mL MTT reagent (Thermo Scientific) diluted in culture media at 37 °C in the last 3 h of GW4869 treatment. The supernatant was then removed from the wells, and the precipitated MTT dye was dissolved in DMSO. The absorbances at 540 nm were recorded with a VICTOR NIVO multimode plate reader (PerkinElmer, Waltham, MA, USA).

*Proteomics*. Proteomic analyses of EVs were performed by the Proteomics and Metabolomics Core of the Cleveland Clinic. EV samples were lysed in a RIPA buffer (containing HALT protease/phosphatase inhibitors (Thermo Fisher), and then sonicated. The lysates were reduced with DTT, alkylated with iodoacetamide, and precipitated with cold acetone prior to tryptic digestion. Lysates were digested with 1 μg trypsin overnight at 37 °C. The digests were evaporated in a SpeedVac and re-suspended in 30 μL formic acid solution (0.1%) for analysis using the LC-MS system (ThermoScientific Exploris 480 mass spectrometer equipped with a Vanquish Neo uHPLC system). A total of 5 μL extract were injected into the column and the peptides were eluted in an acetonitrile/0.1% formic acid gradient at 0.3 μL/min. The peptides were analyzed using data-dependent multitasking, as described before [39]. The SynGO ontology database (version 20231201 [40]), biological processes (BPs), and cellular components (CCs) analysis were used to analyze the identified 429 proteins in neuronal EV samples.

*Study design*. In this study, primary cortical neurons were cultured from embryonic day 18 (E18) mice. Cultured neurons were randomly assigned to treatments or control groups as below:

1. To compare the effects of EV release on intracellular CYFIP2 protein levels via pharmacological inhibition (Figure 2), WT neurons were treated (DIV7) with either the vehicle (DMSO) or 10 µM GW4869. After 24 h, EVs were isolated from the medium, and neurons were collected for further tests.
2. To examine the effects of EV release on intracellular CYFIP2 protein levels via genetic knockout of *Rab27a* (Figure 2), we compared cultured WT and *Rab27a* KO neurons. On DIV8, EVs were isolated from the medium, and neurons were collected for further tests.
3. To examine intracellular CYFIP2 protein level following stimulated EV release (Figure 3), we treated WT neurons with the vehicle (PBS) or 15 µM glutamate on DIV7. At DIV8, EVs were isolated from the medium, and neurons were collected for further tests.
4. To examine EV release and intracellular CYFIP2 protein levels in WT and *Fmr1* KO neurons (Figure 4), EVs were isolated from the culture medium of WT and *Fmr1* KO neurons (DIV8), and neurons were collected.
5. To assess the effect of elevated EV release on intracellular CYFIP2 protein level in *Fmr1* KO neurons (Figure 5), we treated primary *Fmr1* KO neurons with the vehicle (PBS) or 15 µM glutamate on DIV7. At DIV8, EVs were isolated from the medium, and neurons were collected for further tests.

*Statistical analysis*. The detailed statistical analysis used and sample numbers (cell cultures and repeats) are described in figure legends. The data presented are the mean values ± s.e.m. G*Power 3.1 software, and previous studies were used to pre-estimate the sample sizes with modifications. Wells and dishes with cultured primary neurons were grouped randomly. The group and treatment information were labeled anonymously and blinded to the experimenters until data analysis. The normal distribution of collected data was examined using Kolmogorov–Smirnov test. Statistical significances (*p* < 0.05) were assessed with Student’s *t*-test (unpaired, two-sided) using Originpro software (OriginLab, Origin 2025b, Northampton, MA, USA). The variances of data between groups were assessed with Levene’s test (*p* < 0.05) using Originpro (OriginLab). Neurons meeting the pre-established criteria—low viability (<90%), microbial contamination, and morphological abnormalities—were excluded.

## 3. Results

**Figure 1:**
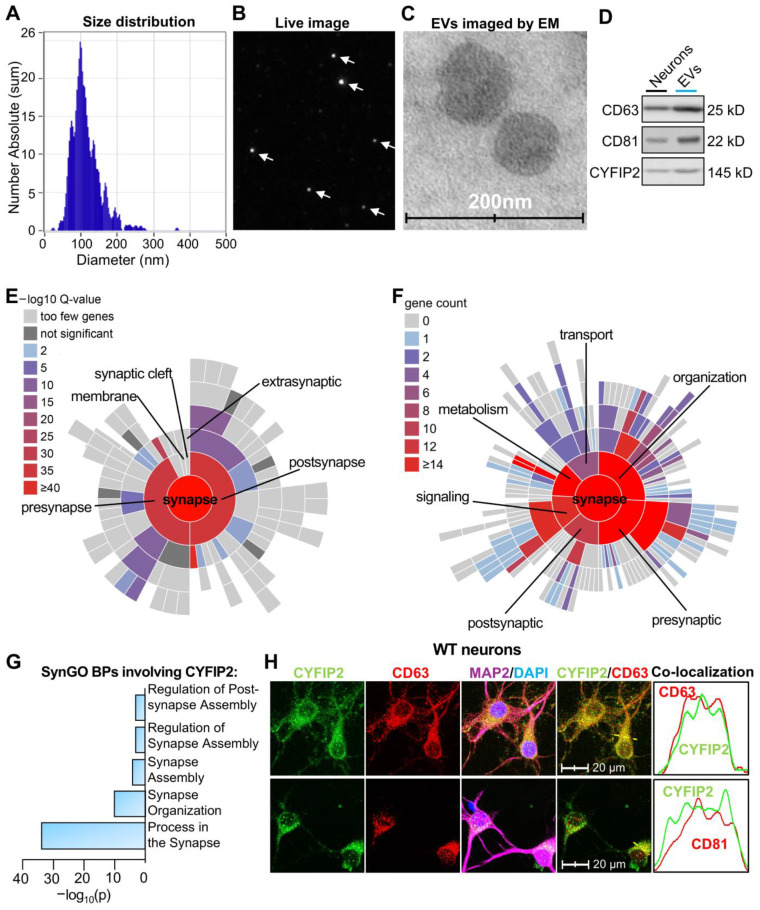
Proteomic analysis revealed CYFIP2 in neuronal EVs. Primary cortical neurons were cultured from WT mice at E18. Fresh medium was added to neurons at DIV7, and extracellular vesicles (EVs) were collected from culture medium at DIV8. (**A**,**B**) Analysis of EVs with nanoparticle tracking system, ZetaView. (**A**) Distribution of collected neuronal EV diameters. (**B**) Live image of EVs from Zetaview. (**C**) EVs imaged by Transmission Electron Microscope (EM). (**D**) Western blot analysis of protein lysates from secreted EVs and donor neurons. (**E**) Sunburst blot displaying SynGO analysis of cellular components (CCs) with each term on a color-coded scale, as indicated. The blot is organized from the parent term, “synapse” in the center, to more refined child terms in the outer shells. (**F**) Sunburst blot shows the SynGO biological processes (BPs) analysis. The number of proteins in each process is indicated on a color-coded scale. The blot is organized from the parent term, “synapse” in the center, to more refined child terms in the outer shells. (**G**) Bar graph shows CYFIP2-involved SynGO BPs of neuronal EVs proteins. (**H**) Immunostaining images show the co-localization of CYFIP2 with EV markers CD63 or CD81, together with neuronal marker MAP2 in neurons. Yellow dashed lines represent the intensity profiles plotted in the right panels. (*n* = 4 independent neuronal cultures). Scale bar, 20 µm. n = 3 independent EV collections for proteomic analysis.

*CYFIP2 and other synaptic proteins are secreted within neuronal EVs*. Recent reports indicated that several synaptic proteins are released by neurons through EVs [30]. To extensively screen synaptic proteins in neuronal EVs, we cultured primary cortical neurons derived from wild-type embryonic mouse pups. On days in vitro 7 (DIV7), the medium was replaced with fresh media. After 24 h (DIV8), EVs were collected through differential ultracentrifugation of the culture medium, as described before [37]. Nanoparticle tracking analysis (ZetaView) revealed diameters of most isolated EVs ranging from 50 to 150 nm, consistent with the size of the exosomes (Figure 1A,B). Transmission electron microscopy (TEM) images with negative staining confirmed the typical spherical morphology of isolated EVs (Figure 1C). EV purity was further validated by the enrichment of EV markers: CD63 and CD81 (Figure 1D). Proteomic analysis of isolated neuronal EVs revealed 429 proteins, including CYFIP2 (Dataset S1). Western blotting further confirmed the presence of CYFIP2 in neuronal EVs (Figure 1D). Synaptic Gene Ontologies (SynGO, version 20231201 [40]) cellular components (CC) analysis revealed that 169 (including CYFIP2) of these 429 EV proteins are located at “synapse”, “presynapse”, and “postsynapse” sites in neurons (Figure 1E, Dataset S2), indicating that synaptic proteins are sorted into and released through EVs. SynGO biological process (BP) analysis revealed that the most enriched synaptic processes these EV proteins are involved in are “organization’, ‘metabolism’ and ‘signaling’ of synapses (Figure 1F). CYFIP2 is extensively involved in synapse-related SynGO BPs, such as “process in the synapse”, “synapse organization”, and “synapse assembly” (Figure 1G). To directly observe the packaging of CYFIP2 into EVs, intracellular precursors of EVs, intraluminal vesicles (ILVs) are labeled with CD63 and CD81 in neurons, and localizations of CYFIP2 in ILVs were observed. Indeed, CYFIP2 localizes to intracellular CD63- or CD81-labeled ILVs (Figure 1H), indicating CYFIP2 proteins are sorted into ILVs, the precursors of EVs before secretion. Consistent with their enrichment in neural EVs shown by the proteomic data, CD81 and CD63 labeled a large amount of ILVs in neurons. Thus, we used CD81 or CD63 as the intracellular ILVs markers for subsequent analyses. In summary, our data indicate that neuronal EVs contain CYFIP2, along with other synaptic proteins that can regulate synapse organization and assembly.

**Figure 2:**
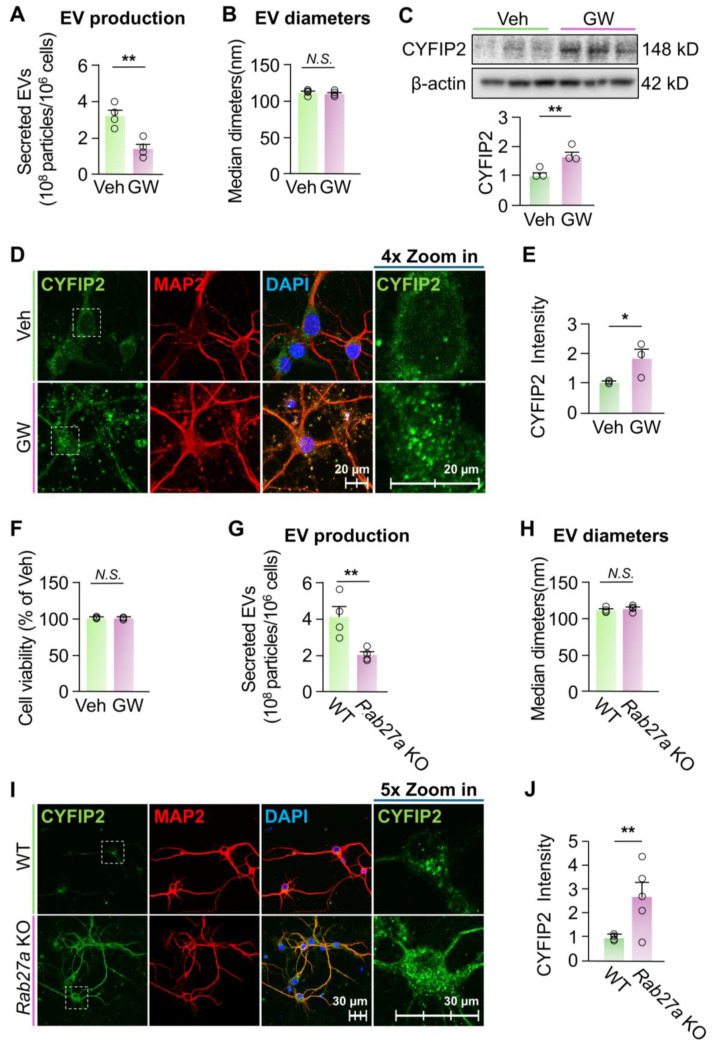
Inhibition of EV release increased intracellular CYFIP2 levels in neurons. (**A**–**F**) Wild-type primary cortical neurons were cultured with fresh medium at DIV7 and treated with vehicle (Veh, DMSO) or GW4869 (GW) at 10 µM for 24 hr. EVs were collected from culture medium at DIV8. (**A**) Bar graph shows the number of EVs collected (*n* = 4). (**B**) Bar graph shows diameters of collected EVs (*n* = 4). (**C**) Intracellular CYFIP2 protein levels were examined with Western blot of protein lysates of treated neurons. Upper: representative blots. Lower: bar graph shows summarized CYFIP2 levels (normalized to Veh) (*n* = 3). (**D**) Veh- and GW4869-treated neurons were immuno-stained with CYFIP2, together with neuronal marker MAP2. Scale bar, 20 µm. CYFIP2 stains in white boxes are shown at higher magnification in the right panels. (**E**) Bar graph shows the summarized CYFIP2 fluorescent intensity of D (normalized to Veh). (**F**) Cell viability tests of neurons treated with Veh or GW4869 (*n* = 3). (**G**–**J**) Primary cortical neurons were cultured from WT or homozygous *Rab27a* KO mice at E18. Fresh medium was added to neurons at DIV7 and EVs were collected from culture medium at DIV8. (**G**) Bar graph shows the number of EVs collected (*n* = 4). (**H**) Bar graph shows diameters of collected EVs (*n* = 4). (**I**) Cultured WT and *Rab27a* KO neurons were immuno-stained with CYFIP2, together with neuronal marker MAP2. Scale bar, 30 µm. CYFIP2 stains in white boxes are shown at higher magnification in the right panels. (**J**) Bar graph shows the summarized CYFIP2 fluorescent intensity of I (*n* = 5). Significance was calculated by student’s *t*-test (unpaired, two-tailed). * *p* < 0.05. ** *p* < 0.01. *N.S.*: no significant difference. β-actin as the loading control. Values reflect the mean ± s.e.m. Each circle represents an independent EV collection in (**A**,**B**,**G**,**H**) or an independent neuronal culture in (**C**,**E**,**F**,**J**).

*Inhibition of EV release increased intracellular CYFIP2 level in neurons*. Next, we investigated whether altered EV secretion could affect the intracellular CYFIP2 level in neurons. First, we treated primary cortical neurons derived from wild-type (WT) mice with DMSO (Veh) or 10 µM GW4869 (GW) for 24 h. GW4869 is a cell-permeable, neutral noncompetitive sphingomyelinase (nSMase) inhibitor [41], which has been widely used to inhibit secretion of EVs in many cell types [41]. GW4869 treatment significantly reduced neuronal EV secretion by 56% compared to neurons treated with Veh (Figure 2A), without significantly affecting diameters of secreted EVs (Figure 2B). To examine the effect of inhibited EV release on intracellular CYFIP2 level, neuronal protein lysates isolated post-GW4869 treatment were subjected to immunoblotting. Neurons treated with GW4869 showed significantly higher levels of CYFIP2 protein compared to neurons treated with the vehicle (Figure 2C). Immunofluorescence confirmed the increased protein level of intracellular CYFIP2 in GW4869-treated neurons (Figure 2D,E). Importantly, more CYFIP2-labeled particles are docked inside the plasma membrane in GW4869-treated neurons, confirming the halted EV-dependent secretion of CYFIP2. GW4869 treatment did not significantly alter neuronal viability (Figure 2F), indicating that the altered EV release and CYFIP2 levels are not caused by cell death.

To further confirm the effect of inhibited EV release on CYFIP2 level, we used a genetic method to inhibit EV release, and examined the intracellular CYFIP2 level. It is well established that the Rab27a protein is critical for the secretion of EVs, such as exosomes, by facilitating MVBs to fuse with plasma membrane, allowing EV secretion [42,43,44,45]. Knockdown of *Rab27a* has been previously used to study the effects of inhibited EV release in neurons and other brain cells [37,46]. Thus, we created a new mouse model with *Rab27a* knockout by inserting a *lacZ* gene, a *neo* gene, and two *Frt* sequences between the exon2 and exon3 of *Rab27a* gene (Figure S1A,B). We cultured primary cortical neurons from *Rab27a* knockout (KO) mice (E18) and knockout of *Rab27a* was validated with ablated Rab27a protein in these neurons (Figure S2). EV secretion of *Rab27a* KO neurons reduced to ~49% of WT neurons (Figure 2G). This reduced EV secretion is consistent with previous studies with knockdown of *Rab27a* [47]. In addition, the mean diameter of the collected EVs shows no significant differences with WT (Figure 2H). Immunofluorescence revealed that the intracellular level of CYFIP2 increased by 168 ± 39% in *Rab27a* KO neurons compared to WT (Figure 2I,J), indicating that genetically inhibiting EV release also resulted in increased intracellular CYFIP2 protein level in neurons. Collectively, our results demonstrated that inhibition of EV secretion leads to accumulation of synaptic protein CYFIP2 within neurons.

**Figure 3:**
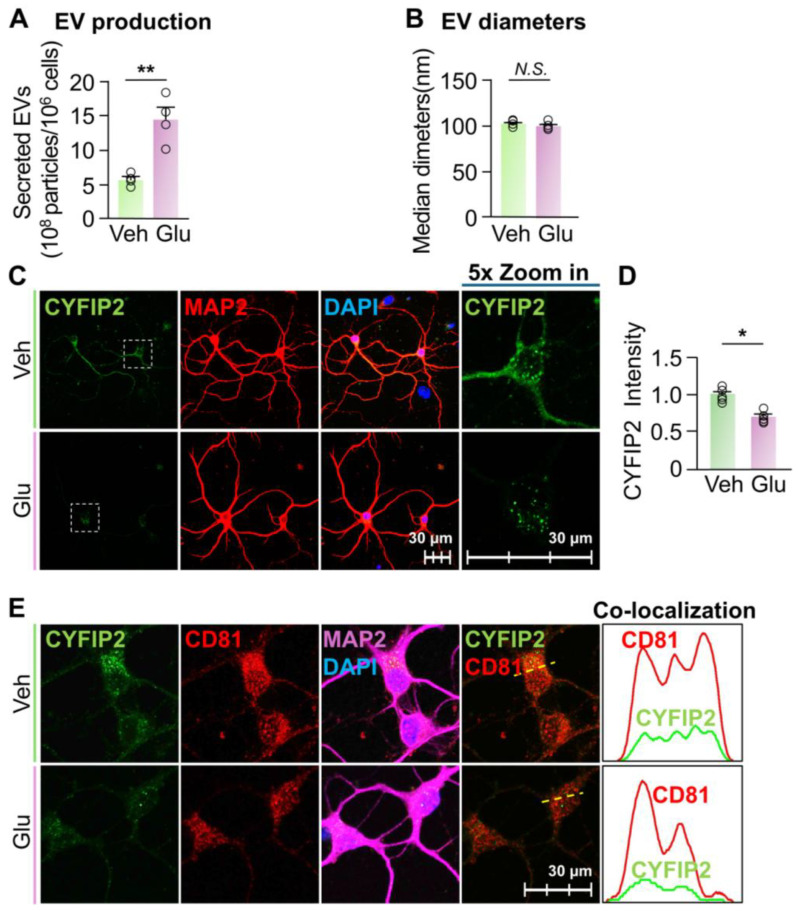
Stimulation of EV release reduced intracellular CYFIP2 level in neurons. Wild-type primary cortical neurons (DIV7) were cultured with fresh medium, and treated with PBS (Veh) or Glutamate (Glu) at 15 µM. EVs were collected from culture medium 24 h after treatment. (**A**) Bar graph shows the number of collected EVs (*n* = 4). (**B**) Bar graph shows diameters of collected EVs (*n* = 4). (**C**) After treatment, neurons were immuno-stained with CYFIP2, together with neuronal marker MAP2. Scale bar, 30 µm. CYFIP2 stains in white boxes are shown at higher magnification in the right panels. (**D**) Bar graph shows the summarized CYFIP2 fluorescent intensity of C (*n* = 5). (**E**) Confocal images show the co-localization of CYFIP2 with EV marker CD81 in neurons. Yellow dashed lines represent the intensity profiles that were plotted in the right panels. Scale bar = 30 µm. Significance was calculated by Student’s *t*-test (unpaired, two-tailed). * *p* < 0.05. ** *p* < 0.01. *N.S.*: no significant difference. Values reflect the mean ± s.e.m. Each circle represents an independent EV collection in (**A**,**B**), or an independent neuronal culture in (**D**).

*Elevated EV secretion reduced the intracellular CYFIP2 level in neurons*. Our results indicated that the inhibition of EV secretion increased the intracellular CYFIP2 protein level in neurons. To establish the causal relationship between EV secretion and intracellular CYFIP2 protein level, we next test whether activating EV secretion can reduce CYFIP2 protein level in neurons. As previously reported, glutamate leads to an influx of extracellular Ca^2+^ into neurons via NMDA and AMPA receptors [34,48,49,50], which elevates the intracellular Ca^2+^ level and significantly promotes neuronal secretion of EVs [34,49,50,51]. Thus, we cultured primary cortical neurons and treated them with either PBS (Veh) or 15 µM glutamate, as previously reported [34,48,49,50]. Consistent with the previous findings, neurons treated with glutamate secreted 151% more EVs compared to neurons treated with Veh (Figure 3A). The size of collected EVs were not significantly changed compared to the control (Figure 3B). Furthermore, after glutamate treatment, there was a significant decrease in intracellular CYFIP2 level (~24 ± 2%) compared to neurons treated with Veh (Figure 3C,D). Consistent with Figure 1G, which shows that CYFIP2 proteins localizes to ILVs before being secreted, glutamate treatment significantly reduced the CYFIP2 in CD81-labeled ILVs (Figure 3E), indicating that glutamate induced the EV-dependent secretion of CYFIP2 protein. In summary, these results demonstrate that the activation of EV release can reduce CYFIP2 protein levels in neurons. Together with the results from inhibited EV secretion, our results indicate that EV-dependent secretion is a crucial pathway regulating intracellular CYFIP2 protein levels in neurons, and dysregulated EV secretion may contribute to the aberrant CYFIP2 accumulation in diseased conditions.

**Figure 4:**
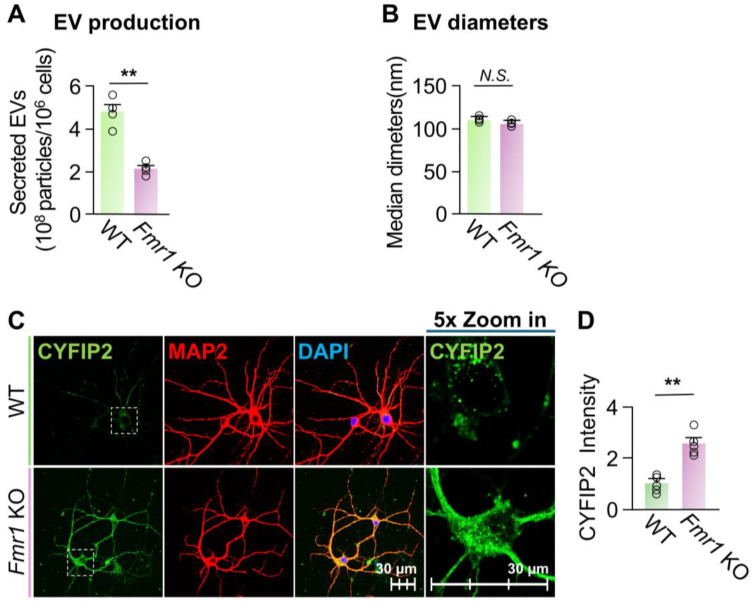
*Fmr1* KO neurons show reduced EV release and increased CYFIP2 level. Primary cortical neurons were cultured from WT or *Fmr1* KO mice at E18. Fresh medium was added to neurons at DIV7 and EVs were collected from culture medium at DIV8. (**A**) Bar graph shows the number of collected EVs (*n* = 4). (**B**) Bar graph shows diameters of collected EVs (*n* = 4). (**C**) Cultured WT and *Fmr1* KO neurons were immuno-stained with CYFIP2, together with neuronal marker MAP2. Scale bar, 30 µm. CYFIP2 stains in white boxes are shown at higher magnification in the right panels. (**D**) Bar graph shows the summarized CYFIP2 fluorescent intensity of C (*n* = 5). Significance was calculated by Student’s *t*-test (unpaired, two-tailed). ** *p* < 0.01. *N.S.*: no significant difference. Values reflect the mean ± s.e.m. Each circle represents an independent EV collection in (**A**,**B**), or an independent neuronal culture in (**D**).

Fmr1 KO neurons show reduced EV secretion. Previous studies have shown that, in diseased conditions, EV secretions of many cell types are dysregulated [52,53]. Cells from patients with FXS have increased CYFIP2 protein levels [11]. Additionally, studies have shown that neurons of *Fmr1* KO mice, an FXS mouse model, have an increased number of vesicles docked inside the plasma membrane, instead of being released, compared to WT neurons [54]. Hence, to determine whether this increased intracellular CYFIP2 protein in FXS neurons is due to altered EV secretion, we first compared WT and *Fmr1* KO neurons’ capacities of EV secretion. Our results revealed that *Fmr1* KO neurons secreted only approximately 46% of the amount of EVs produced by WT neurons (Figure 4A), which is consistent with the reported increased number of vesicles docked inside plasma membrane [54]. In addition, there was no significant difference between the sizes of collected EVs from WT and *Fmr1* KO neurons (Figure 4B). Immunofluorescence confirmed that *Fmr1* KO neurons exhibited a 155 ± 31% increase in intracellular CYFIP2 protein level compared to WT neurons (Figure 4C,D). In summary, our results demonstrated that, compared to WT neurons, *Fmr1* KO neurons show reduced EV secretion and elevated the intracellular CYFIP2 protein level, suggesting that reduced EV secretion may contribute to increased CYFIP2.

**Figure 5:**
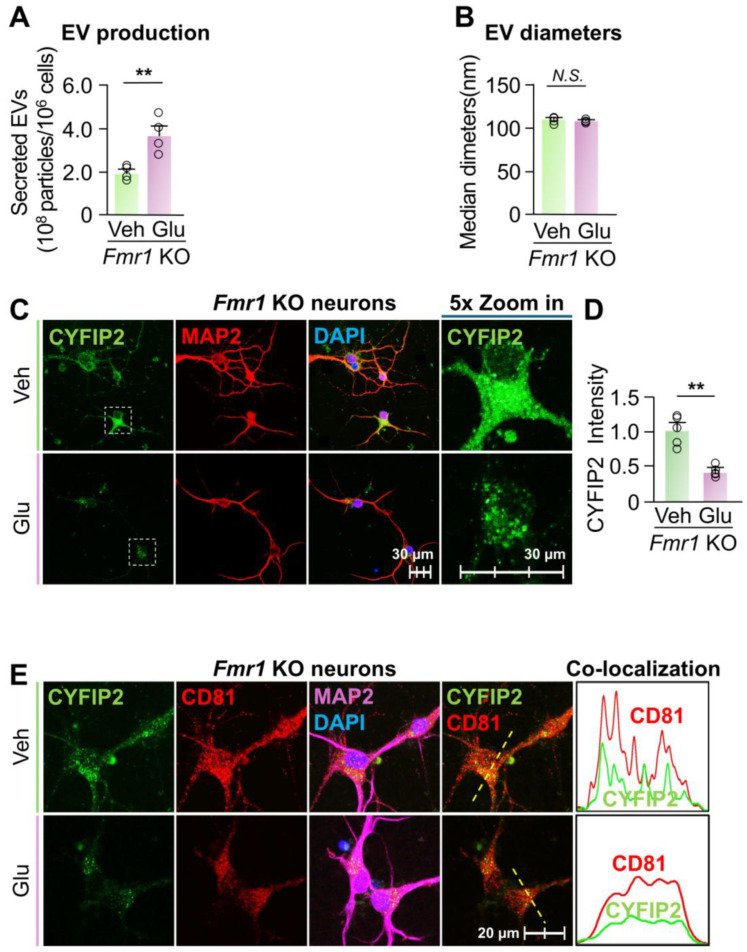
Activation of EV release reduced CYFIP2 level in *Fmr1* KO neurons. Primary cortical neurons were cultured from *Fmr1* KO mice at E18 and treated with PBS (Veh) or Glutamate (Glu) at 15 µM. EVs were collected from culture medium 24 h after treatment. (**A**) Bar graph shows the number of collected EVs (*n* = 4). (**B**) Bar graph shows diameters of collected EVs (*n* = 4). (**C**) After glutamate treatment, neurons were immuno-stained with CYFIP2, together with neuronal marker MAP2. Scale bar, 30 µm. CYFIP2 stains in white boxes are shown at higher magnification in the right panels. (**D**) Bar graph shows the summarized CYFIP2 fluorescent intensity of C (*n* = 5). (**E**) Confocal images show the co-localization of CYFIP2 with EV marker CD81 in neurons after glutamate treatment. Yellow dashed lines represent the intensity profiles that were plotted in the right panels. Scale bar, 20 µm. Significance was calculated by Student’s *t*-test (unpaired, two-tailed). ** *p* < 0.01. *N.S.*: no significant difference. Values reflect the mean ± s.e.m. Each circle represents an independent EV collection in (**A**,**B**), or an independent neuronal culture in (**D**).

*Activation of EV secretion reduced the intracellular CYFIP2 level in Fmr1 KO neurons*. Given the significant increase in the intracellular level of CYFIP2 protein within *Fmr1* KO neurons, we next investigated whether stimulating EV release could reduce CYFIP2 accumulation. To this end, cultured *Fmr1* KO cortical neurons were treated with Veh or 15 µM glutamate. Glutamate treatment increased EV secretion by approximately 88% without significantly affecting the size of the collected EVs (Figure 5A,B). Consistently, glutamate treatment reduced the intracellular level of CYFIP2 protein by 53 ± 6% in *Fmr1* KO neurons compared to the vehicle (Figure 5C,D). Furthermore, glutamate-treated neurons show reduced colocalization of CYFIP2 with CD81, indicating reduced CYFIP2 protein docked in ILVs and enhanced EV secretion (Figure 5E). In conclusion, our results demonstrate that activating EV secretion reduces intracellular CYFIP2 levels, further supporting that the accumulation of CYFIP2 protein in FXS neurons is due to the compromised EV release.

## 4. Discussion

EVs, especially small EVs, such as exosomes, are defined by size and biogenesis from MVBs, and have been increasingly recognized as active vehicles for intercellularly transferring proteins and RNAs in the brain [25,26,27,28]. Our proteomic analysis shows that neurons package numerous proteins into EVs, including synaptic regulators such as CYFIP2. Two independent manipulations: chemical inhibition of EV release and genetic reduction in secretion using a *Rab27a* loss-of-function model, produced significant elevations of intracellular CYFIP2. Conversely, enhancing EV secretion with glutamate lowered intracellular CYFIP2 in neurons. Together, these findings support a model in which neurons use EV secretion to tune intracellular levels of specific synaptic proteins. When outward flux is constrained, proteins such as CYFIP2 accumulate inside neurons.

These observations are consistent with prior reports that EV cargo loading is regulated and functionally consequential in the nervous system. During inflammation, selective secretion of EVs containing miR-155 has been linked to complementary sequence motifs and FMR1-associated machinery [55]. Other studies show that boosting EV delivery of miR-146a ameliorates synaptic dysfunction and promotes synaptogenesis [56], while hippocampal EVs collected after an experimental stroke exhibited reduced synapse-associated proteins (e.g., Synaptotagmin, PSD-95), which is associated with diminished spine density and altered trophic signaling [57]. Our results show that the synaptic protein CYFIP2 is contained in neuronal EVs, supporting the concept that neuronal EVs carry synaptic protein cargos that are critical for synaptic functions. By regulating the secretion of CYFIP2 and other synaptic protein cargos, neurons can modify their own synapses and may potentially influence synapses of other neurons receiving these EVs. In addition to CYFIP2, other synaptic proteins such as PSD-95, VGLUT1, and Synaptotagmin are also packaged into EVs and secreted by neurons [31,32]. Importantly, recipient neurons receiving these neuronal EVs indicate increased intracellular levels of these synaptic proteins [31,32], consistent with our results of CYFIP2 showing that the amount of synaptic proteins secreted through EVs is sufficient to effectively influence their intracellular levels in neurons.

Importantly, EV-dependent secretion of synaptic proteins may play important roles in FXS pathophysiology. Neurons from FXS patients show overabundant excitatory synapses with immature morphology, which are associated with autistic behaviors, such as hypersensitivity and social deficits [1,3]. CYFIP2 participates in pre- and postsynaptic processes that coordinate actin remodeling, translation control, and synapse stabilization [18,19]. Synaptic morphology and structure are critically supported by F-actin, the most important and abundant synaptic cytoskeletal protein [10]. Cofilin1 binds to the pointed ends of F-actin [58] and depolymerizes F-actin, thereby disassembling F-actin and eliminating synapses [59,60]. In FXS neurons, elevated (Serine 3)-phosphorylation inactivates Cofilin1, preventing Cofilin1’s binding to F-actin, suppressing F-actin depolymerization and synaptic elimination, which critically leads to the overabundant immature synapses and FXS symptoms [10,61,62,63,64]. In heathy neurons, CYFIP2 binds with FMRP protein, the expressing product of the *Fmr1* gene [15,24]. However, in FXS neurons, the CYFIP2 level is increased [11]. The absent FMRP cannot request these elevated CYFIP2, releasing them to form Rac1-WAVE complex, which directly phosphorates (inactivate) Cofilin1, suppresses synaptic elimination, and thereby leads to overabundant immature synapses [24,65,66]. Overexpressing CYFIP2 in WT hippocampal neurons increased the density of the immature synapses, phenotype-copying FXS neurons, which can be further identified as an important role of CYFIP2 in FXS pathology [16].

Our data indicate that reduced EV release in FXS neurons is associated with intracellular retention of CYFIP2, suggesting a mechanism for the excess of immature dendritic spines in FXS: when EV-mediated “export” is impaired, CYFIP2 accumulates, potentially dysregulating actin-dependent remodeling and leading to excessive excitatory synapses. These results identify a previously unrecognized EV release-dependent mechanism underlying the synaptic deficits of FXS. In addition to CYFIP2, 169 synaptic proteins are identified in neuronal EVs. FXS neurons exhibit reduced EV release, potentially altering the intracellular levels of all these 169 EV proteins. Although we focused on CYFIP2 protein in this work, other identified synaptic proteins in EVs may also play important roles in regulating synaptic density and functions. Thus, FXS synaptic deficits may arise from the synergistic effects of many dysregulated synaptic proteins.

## 5. Conclusions

In summary, our findings first revealed that CYFIP2 and other synaptic proteins are secreted by neurons through EVs. Then, we demonstrated that inhibiting EV secretion increased while elevated EV secretion reduced the intracellular CYFIP2 level in neurons, indicating that the amount of CYFIP2 protein in EVs is sufficient to significantly affect its intra-neuronal level. FXS neurons indicated an enhanced CYFIP2 level but reduced EV secretion ability. We further elucidated that activating EV secretion could significantly reduce the intracellular CYFIP2 level in *Fmr1* KO neurons. Thus, our study identifies EV-mediated secretion as a critical finetune for the homeostasis of the intracellular CYFIP2 level, and implicates impaired EV release as a potential contributor to synaptic abnormalities in FXS.

## Data Availability

All data used to support this study are available from the corresponding author upon request. Reagents and all other data are available from the corresponding author upon reasonable request. The datasets have been submitted as supplemental materials of this paper.

## Supplementary Materials

The following supporting information can be downloaded at: https://www.mdpi.com/article/10.3390/biomedicines13102518/s1. Figure S1: Generation of *Rab27a KO.* Figure S2: Genotyping of *Rab27a* KO and Dataset S1–S2.
