# Supplementary material for: Extracellular Vesicles-Dependent Secretion Regulates Intracellular CYFIP2 Protein Homeostasis in Cortical Neurons
Source: Biomedicines. 2025 Oct 15;13(10):2518. doi: 10.3390/biomedicines13102518 (PMC12561013; doi:10.3390/biomedicines13102518)
Supplement: Supplementary file 1 [file biomedicines-13-02518-s001.zip › CYFIP2 Supplementary Figures Final.pdf]

Supplementary Figure 1

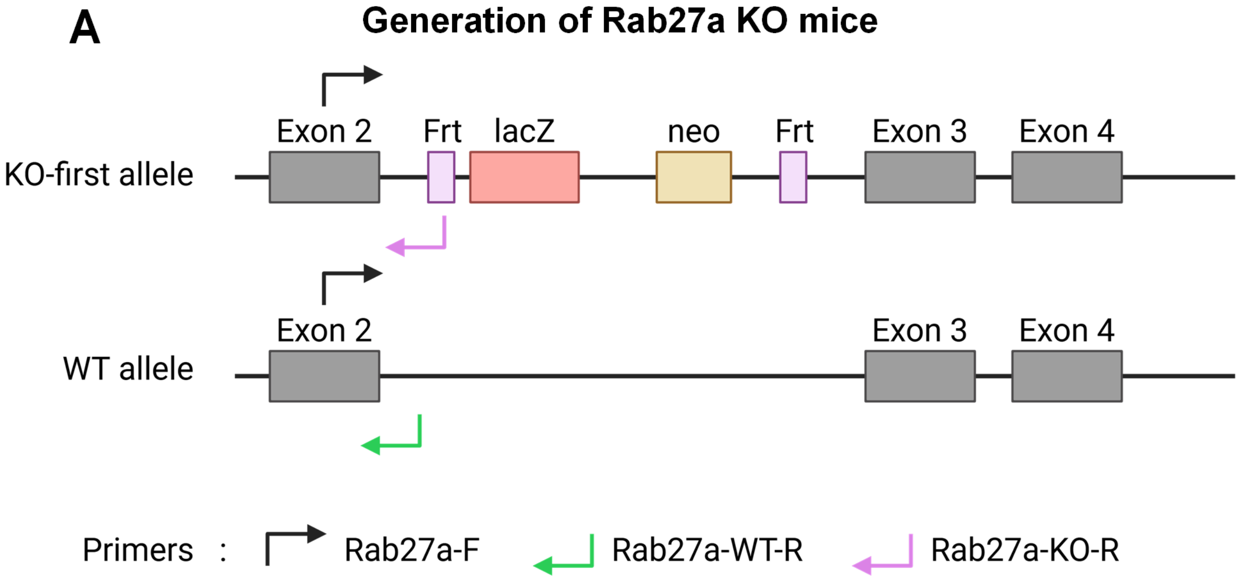

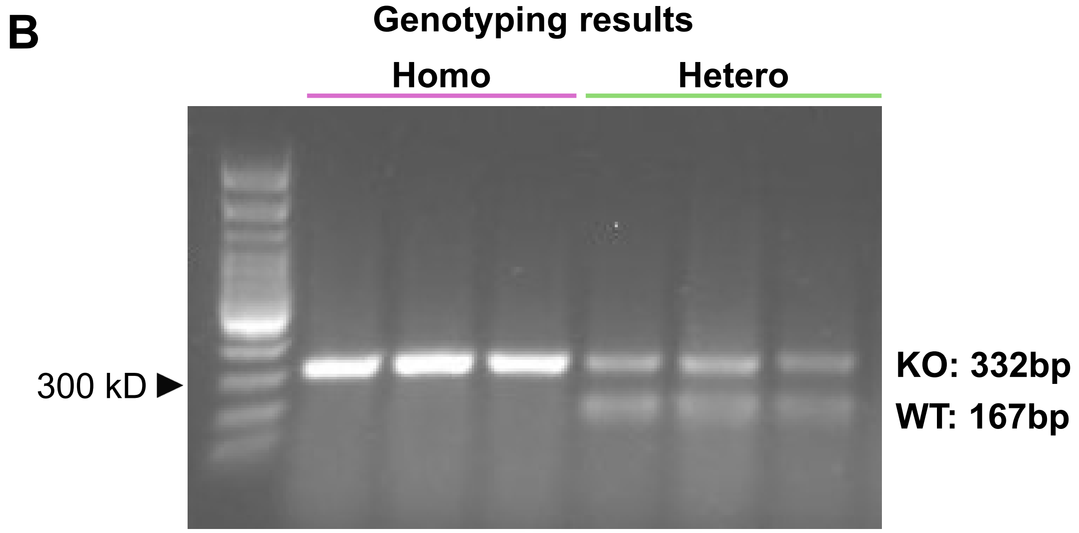

Suppl Figure 1. Generation of Rab27a KO mice. (A) Schematic illustration of generation of Rab27a KO mice. A lacZ gene, a neo gene, and two Frt sequences were inserted between exon2 and exon3 of Rab27a gene in the genome of WT mice, leading to a longer KO allele (332bp) vs WT allele (167bp) in PCR. (B) Representative DNA gel of genotyping.

Supplementary Figure 2

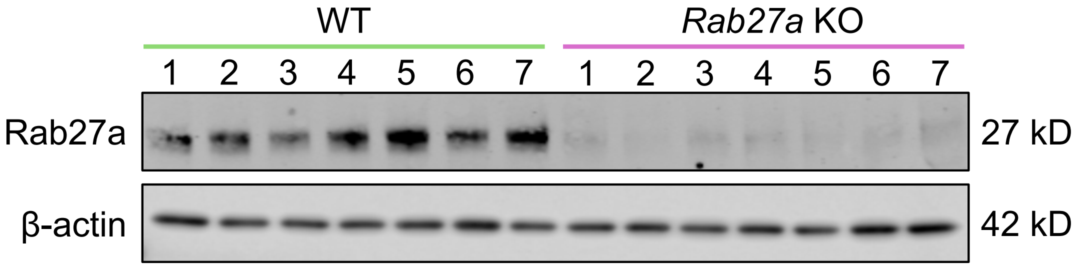

Suppl Figure 2. Validation of Rab27a knockout in cultured neurons. Primary cortical neurons were prepared from wild-type (WT) and homozygous Rab27a knockout (KO) mice at E18. At DIV8, whole-cell lysates were prepared and Rab27a protein levels were assessed by Western blot. Numbers above the lanes denote independent culture batches
